# Predicting 30-day hospital readmissions using ClinicalT5 with structured and unstructured electronic health records

**DOI:** 10.1371/journal.pone.0328848

**Published:** 2025-09-02

**Authors:** Sanjib Raj Pandey, Joy Dooshima Tile, Mahdi Maktab Dar Oghaz

**Affiliations:** 1 The Royal Marsden NHS Foundation Trust, Digital Services, London, United Kingdom; 2 Faculty of Science and Engineering, Anglia Ruskin University, Cambridge, United Kingdom; Shanghai Jiaotong University: Shanghai Jiao Tong University, CHINA

## Abstract

Hospital readmission prediction is a crucial area of research due to its impact on healthcare expenditure, patient care quality, and policy formulation. Accurate prediction of patient readmissions within 30 days post-discharge remains a considerable challenging, given the complexity of healthcare data, which includes both structured (e.g., demographic, clinical) and unstructured (e.g., clinical notes, medical images) data. Consequently, there is an increasing need for hybrid approaches that effectively integrate these two data types to enhance all-cause readmission prediction performance. Despite notable advancements in machine learning, existing predictive models often struggle to achieve both high precision and balanced predictions, mainly due to the variability in patients’ outcome and the complex factors influencing readmissions. This study seeks to address these challenges by developing a hybrid predictive model that combines structured data with unstructured text representations derived from ClinicalT5, a transformer-based large language model. The performance of these hybrid models is evaluated against text-only models, such as PubMedBERT, using multiple metrics including accuracy, precision, recall, and AUROC score. The results demonstrate that the hybrid models, which integrate both structured and unstructured data, outperform text-only models trained on the same dataset. Specifically, hybrid models achieve higher precision and balanced recall, reducing false positives and providing more reliable predictions. This research underscores the potential of hybrid data integration, using ClinicalT5, to improve hospital readmission prediction, thereby improving healthcare outcomes through more accurate predictions that can support better clinical decision making and reduce unnecessary readmissions.

## Introduction

Improving healthcare outcomes is a global priority, with unnecessary and unplanned hospital readmissions presenting a significant challenge. These readmissions are costly, put pressure on healthcare systems, and negatively impact patients and caregivers. They are also widely recognized as a key indicators of healthcare quality. In the United States (US), about 20% of patients are readmitted within 30 days after discharge [1]. In the United Kingdom (UK), emergency readmission rates reached 15.5% in 2020/21, despite a decrease in overall admissions during the COVID-19 pandemic [2,3]. High readmission rates often reflect deeper issues within the healthcare system.

**Fig 1:**
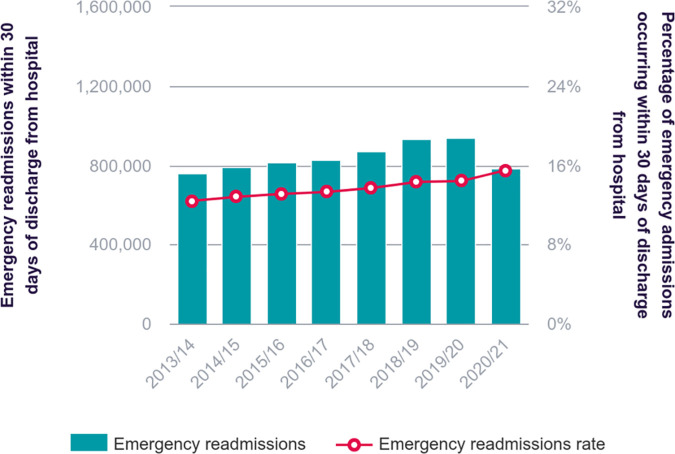
The emergency readmission rates in the United Kingdom Source: https://www.nuffieldtrust.org.uk/resource/emergency-readmissions, Copyright Nuffield Trust & the Health Foundation. Access on 22/12/204.

To address the growing issue of readmissions, the Hospital Readmissions Reduction Program (HRRP) was introduced under the Affordable Care Act (ACA) in the US. This program penalizes U.S. hospitals with higher-than-expected 30-day readmission rates, [4,5], emphasizing the need to understand and reduce unplanned readmissions [6]. However, identifying patients at high risk of readmission remains a complex task, influenced by factors such as individual health conditions, social determinants of health (SDOH), and systemic healthcare processes. In the U.K., emergency readmissions cost the National Health Service (NHS) an estimated £1.6 billion each year [7], while in the U.S., the average cost of a readmissions was $16,300 in 2020, a 12.4% increase compared to initial admission costs [8]. Fig 1 illustrates the trends in 30-day emergency readmission in the UK over time.

Recent advances in Artificial Intelligence (AI) and Machine Learning (ML) provide new opportunities to addressing this challenge. The emergence of powerful ML models facilitates resolution of complex challenges such as hospital readmission, as these algorithms can detect patterns that may be too complex and subtle for traditional statistical methods. These ML models can even assess unstructured data such as doctors’ notes, and generate risk scores for readmission, which aids (as a decision support system) clinicians to identify high-risk patients and target interventions more effectively [9].

Another advantage of these models is that they can be easily updated with new data as outcomes become available, allowing them to continuously learn, adapt to new data, and improve their predictive ability over time. Leveraging these techniques can improve patient outcomes, reduce financial burdens linked to high readmission rates, and enhance the quality and cost-effectiveness of care.

## Problems

Hospital readmissions represent a significant cost to the healthcare system and present multiple challenges to patient care. Many readmissions can be prevented through better care coordination and discharge procedures [6]. Traditionally, predicting readmission risk relied on simple models [10–12], which which often overlooked the complex factors influencing readmissions. For example, models like the LACE Index [10] are useful but often lack accuracy across different patient groups and do not include unstructured clinical notes, which can provide invaluable critical insights. Recent research has focused on combining structured and unstructured data to improve readmissions prediction accuracy. However, traditional Natural Language Processing (NLP) models struggle with long clinical text, leading to data loss and reduced predictive accuracy [13]. There is a clear need for methods that can fully use clinical notes without losing valuable data. At the same time, advanced models such as Large Language Models (LLMs) often require high computational power, which may not be practical in some research settings. Our approach integrates unstructured data while taking these limitations into account.

## Proposed solution

This study presents a novel approach for processing clinical notes by dividing them into 200-word segments, which are then analyzed using the ClinicalT5 Large Language Model [14]. These text segments are combined with structured patient data to predict patient hospital readmissions. The method aims to balance computational resource constraints with the richness of clinical data and explores whether segmenting clinical text can improve the accuracy of readmission predictions.

The study addresses the following research questions:

1. How does integrating 200-word text segments, processed using ClinicalT5, with structured healthcare data, enhance hospital readmission predictions?
2. How does this segmented approach compare in terms of prediction accuracy and scalability to traditional methods that use truncated clinical texts?

This approach offers a practical step toward fully leveraging unstructured clinical data within resource limitations. By evaluating its feasibility and benefits in a controlled setting, the study aims to support border applications of LLMs and other advanced NLP techniques in healthcare. Specifically, it investigates the impact of ClinicalT5 on predicting hospital readmission by integrating structured Electronic Health Record (EHR) data with free-text clinical notes. This includes using embeddings from clinical notes and diagnoses extracted through a fine-tuned version of ClinicalT5. By segmenting clinical notes into smaller, manageable chunks, the study seeks to preserve the richness of the data and while assessing the effectiveness of combining structured and unstructured data for predicting 30-day readmissions.

## Main objectives

The primary objectives of this study are as follows:

1. To fine-tune the ClinicalT5 on segmented unstructured clinical notes, using 200-word chunks, in order to effectively capture contextual clinical representations.
2. To develop a Hybrid Predictive Model that integrates structured healthcare data with ClinicalT5-derived embeddings, with the aim of enhancing the prediction of 30-day hospital readmissions.
3. To fine-tune PubMedBERT on the same clinical notes to establish a comparative baseline for evaluating the effectiveness of the proposed hybrid model.
4. To evaluate and compare the performance of the hybrid model and the PubMedBERT-based model using standard predictive metrics, including accuracy, precision, recall, and the area under the receiver operating characteristic curve (AUROC), particularly focusing on the limitations of relying solely on truncated unstructured text.

## Literature review

The rise of machine learning has driven a technological transformation across industries, including healthcare, where predictive models have largely relied on structured data such as demographic information, laboratory results, and vital signs. However, free-form clinical notes contain rich, underutilized information that can further enhance predictive performance. Hospital readmission prediction is a critical application where the potential of unstructured clinical data remains largely underutilized.This review examines recent developments in hospital readmission prediction, with a focus on integrating structured data—such as demographics and vital signs with unstructured data from clinical notes. Special attention is given to the emerging role of LLMs in improving predictive accuracy. Despite significant progress, a notable gap remains in effectively combining LLMs with structured data, which this review seeks to explore and address.

### Structured data

Structured data from sources like Electronic Health Records (EHRs) and administrative claims have played a key role in enhancing machine learning’s predictive capabilities in healthcare. Due to its fixed structure, this data is relatively straightforward to analyze, making it suitable for rule-based methods, traditional statistical models, and machine learning techniques.

### Traditional statistical models

Historically, hospital readmission prediction models have mainly used methods like logistic regression, which are simple, interpretable, and effective for binary classification [15,16]. One example is the HOSPITAL score, a clinical rule-based tool designed to predict 30-day readmissions using patient and administrative data collected prior to discharge. The score incorporates seven independent factors to identify high-risk patients [11]. Similarly, the LACE index, introduced in 2010 [10], is another clinical rule-based developed to quantify the risk of readmission or death following discharge. It achieved a C-statistic of 0.684 after external validation. The LACE index uses four variables: Length of Stay, Acuity of Admission, Patient Comorbidity, and Emergency Department use, to predict readmission risk. Despite its widespread use, the LACE index has been criticized for its moderate discriminative ability [17]. Like the LACE index, the HOSPITAL score also demonstrates only moderate discriminative power and does not fully utilize the rich patient data available in Electronic Health Records.

Another widely used predictive model based on logistic regression is the Patient at Risk of Readmission within 30 Days (PARR-30) model, developed by [12]. This model, which achieved a C-statistic of 0.70, utilizes a broader range of variables, including patient demographics, previous hospital admissions, and clinical data. However, it still heavily relies on administrative data, which limits its predictive accuracy. Despite these limitations, the PARR-30 model is actively used by the NHS for policy-making, demonstrating its practical value.

Logistic Regression (LR) continues to be one of the most commonly used methods for predicting hospital readmissions. A review by [18]found that 68% of the studies employed LR or other regression techniques as their primary approach. These models typically depend on manually derived features from patient data, such as demographics, comorbidities, and healthcare utilization patterns. However, such models often exhibit only moderate discriminative ability in predicting hospital readmissions [19].

In a 2019 study, [20] applied the HOSPITAL score and LACE index to a dataset containing medical claims data from over 100,000 patients in the Geisinger Health System. Both methods achieved an area under the receiver operating characteristic curve (AUC) score of 0.60, with the HOSPITAL score performing slightly better. In another study, [21], developed a logistic regression model using health insurance claims data from 138,222 hospitalized adults in Switzerland. This model incorporated variables such as pharmacy-based cost groups, emergency visits, and outpatient costs. This model also achieved an AUC score of 0.60, indicating limited discriminative ability, only slightly better than chance (0.5). hese findings further suggest that predictive models developed using Electronic Health/Medical Records data tend to offer better performance than those based on insurance claims or other administrative data [20,22].

### Machine learning models

On the other hand, Machine Learning (ML) models have shown promise in improving the accuracy of readmission predictions. The types of machine learning (supervised, unsupervised, and reinforcement learning), along with their various applicable algorithms, are discussed and presented by Pandey et al. [23]. In a review of 9 studies, [16] evaluated the performance of logistic regression and ML models and found that ML models generally outperformed logistic regression in predicting 30-day all-cause hospital readmissions, with deep learning models performing the best.However, they observed that tree-based and kernel-based methods did not offer significant improvements. The authors concluded that while ML models outperform traditional methods in predicting hospital readmissions, challenges such as model interpretability and integration into clinical workflows remain unresolved.

Conversely, Min et al. [20] compared traditional ML models (e.g., logistic regression, random forest) with deep learning techniques (e.g., CNN, RNN) for predicting readmission risk, using both knowledge-driven and data-driven features. Despite the complexity of deep learning models, the Gradient Boosting Decision Tree (GBDT) achieved the highest AUC (0.653), while deep learning methods did not show significant improvement. This raises questions about the added value of deep learning in certain clinical prediction tasks, especially when simpler models provide similar performance.

A range of studies have applied machine learning models to structured data but very few have achieved consistently high metrics. Typically, when a model achieves high recall, t is often accompanied by low precision, and vice versa. For instance, Lo et al. [24] developed predictive models for 14-day unplanned readmissions, and among these, CatBoost delivered the best performance, achieving an AUROC of 0.9909 after feature selection.However, the model also exhibited a moderate sensitivity of 0.56. While not necessarily poor, this suggests a potential class imbalance, with the Receiver Operating Characteristic (ROC) curve potentially biased towards the positive class. A similar issue was observed in the study by [25], which aimed to improve early Intensive Care Unit (ICU) readmission prediction. Using Extreme Gradient Boosting (XGBoost) and structured data from the MIMIC-III dataset, they achieved an AUROC of 0.92 ± 0.03, significantly outperforming previous state-of-the-art models (AUROC ranging from 0.66 to 0.78). However, their model reported a specificity of 0.99 and a recall of only 0.40, again highlighting the challenge of class imbalance.

To address the issue of class imbalance, Yu et al. [26] employed a modified weight-boosting algorithm combined with a stacking method to predict hospital readmissions. The authors used advanced feature engineering techniques to manage the high dimensionality and sparsity of medical codes. Their model was trained and validated on a large nationwide healthcare dataset from China, comprising inpatient administrative data, and achieved a high recall of 0.891, outperforming benchmark models. Despite these promising results, the proposed approach may face practical limitations due to its high computational demands, particularly when applied to large datasets. Furthermore, successful implementation requires careful feature engineering and parameter tuning, demanding a high level of expertise in machine learning.

Magboo et al. [27] compared the performance of three models: Random Forest, Adaptive Boosting, and K-Nearest Neighbors for predicting hospital readmissions among diabetic patients. The study incorporated Local Interpretable Model-agnostic Explanations (LIME) method to provide visual interpretations of the models’ predictions. All three models achieved high accuracy, exceeding 92%, and the LIME outputs offered clinically meaningful insights into the factors driving predictions. However, the study was limited in scope, focusing exclusively on diabetic patients and relying on simulated rather than real-world hospital data, which may limit its generalisability and practical applicability.

Liu et al. [28] introduced an Optimal Variational Bayesian Logistic Regression (OVBLR) model enhanced with a Salient Feature Estimation (SFE) strategy, referred to as OVBLR-SFE, to address the limitations in existing predictive methods by prioritizing accuracy over interpretability.he model demonstrated strong performance and stability across four benchmark medical datasets from the UCI repository, as well as in a real-world application predicting intensive care unit (ICU) readmissions for liver transplant patients. It achieved an average classification accuracy of 90.10% on the UCI datasets and 88.11% on the ICU readmission task. Despite these promising results, the model encountered scalability issues due to its high computational requirements when applied to larger datasets.

Recent review studies by [29] found that, although traditional statistical models are still widely used, machine learning (ML) and deep learning (DL) techniques have demonstrated promising performance in predictive healthcare tasks.

### Deep learning

Jamei et al. [30] investigated the use of artificial neural networks (ANNs) to predict 30-day hospital readmissions, utilizing data from over 300,000 hospital stays in California. The ANN was trained on features derived from electronic health records (EHRs) and social determinants of health, and it outperformed traditional models, including the LACE index. Specifically, it achieved a precision of 0.24 in identifying high-risk patients, representing a 20% improvement over LACE’s precision of 0.20. However, the model faced limitations in generalizability to other patient populations and encountered implementation challenges due to its lack of interpretability.

Barbieri et al. [31] compared several deep learning architectures including attention mechanisms, recurrent layers, neural ordinary differential equations (ODEs), and medical concept embeddings using the MIMIC-III dataset [32]. Among the models evaluated, recurrent neural networks (RNNs) with time-dynamic code embeddings generated by neural ODEs achieved the highest average precision of 0.331, with an AUROC of 0.739 and an F1-score of 0.372. The study found that attention-based models offer improved interpretability with only a minimal reduction in accuracy. Interpretation of the attention model indicated that patients at higher risk of readmission often presented with infectious complications, chronic or progressive conditions, or required non-standard medical care. However, the study did not incorporate unstructured clinical notes, limiting the scope of the analysis. Machine Learning models have come a long way in using structured data for hospital readmission prediction, however, structured data alone is not a complete representation of a patient’s condition.

### Unstructured data

The evolution of predictive models has increasingly highlighted the value of clinical notes, such as radiology reports and discharge summaries [29]. Natural Language Processing (NLP) techniques are capable of analyzing clinical notes, admission and discharge summaries, and other text-based data to identify risk factors and patterns that may not be captured by structured data alone. Sheikhalishahi et al. [33] reviewed the application of various NLP methods in clinical research, emphasizing the need for NLP to evolve beyond text extraction to an understanding of clinical concepts. Their study identified gaps in the use of NLP, particularly regarding the limitations of text extraction, entity recognition in isolation, and reliance on shallow classification methods.

Wu et al. [34] reviewed the growing use of deep learning (DL) in clinical Natural Language Processing (NLP), noting that studies in this area have been increasing rapidly, with Recurrent Neural Networks (RNNs) and word2vec embeddings being among the most commonly employed methods. The primary tasks addressed include text classification, named entity recognition, and relation extraction. However, they also observed that deep learning models do not always outperform simpler models, as demonstrated by Christodoulou et al. [35]. While RNNs are effective, they struggle to capture long-range dependencies in sequences [13,36], particularly with longer sequences. As an alternative, the transformer architecture, introduced by [37], relies entirely on attention mechanisms and eschews recurrent layers. By using multi-headed self-attention, transformers can process all positions in the input and output sequences in parallel, offering improved computational efficiency.

### Large language models

Large Language Models (LLMs) have revolutionized NLP by significantly enhancing the understanding and generation of textual data. Trained on vast amounts of text, these models are capable of performing a wide range of downstream tasks. Based on the transformers architecture, Bidirectional Encoder Representations from Transformers (BERT), developed by Delin J et al. [38], was introduced to capture bidirectional context, which is essential for understanding the relationships between words in a sentence.

Despite the advancements of Large Language Models (LLMs) in processing large text corpora, domain-specific models are still more beneficial in many contexts. Alsentzer et al. [39] applied BERT to clinical notes and released publically available clinically trained BERT models. Similarly, Huang et al. [40] introduced ClinicalBERT, adapted for readmission prediction and pre-trained on longer sequence lengths. ClinicalBERT demonstrated superior predictive performance compared to baseline models, including Bidirectional Long Short-Term Memory (Bi-LSTM) and BERT, when using notes from early patient admissions (AUROC: 0.674) and discharge summaries (AUROC: 0.714). Nazyrova et al. [41] further demonstrated the potential of domain-specific LLMs to enhance predictive accuracy for hospital readmissions. Their study explored BERT variants such as BioBERT [42], SciBERT [43] and ClinicalBERT [40] in predicting 30-day readmissions for elderly patients. Their findings revealed that domain-specific models, particularly SciBERT, outperformed general LLMs in medical contexts. Using the MIMIC-IV dataset, SciBERT improved the AUROC for readmission prediction from 0.714 to 0.735. Although LLMs excel at processing and understanding clinical notes, their integration with structured data remains a largely unexplored area.

### Combining structured and unstructured data

While both structured and unstructured data offer distinct advantages in clinical predictive modeling, emerging evidence suggests that the most effective approach combines both data types. Studies have consistently demonstrated that models integrating both structured (e.g., lab results, medications) and unstructured (e.g., clinical notes, imaging reports) data from electronic health records (EHRs) outperform those relying on a single data source. This approach significantly enhances the accuracy of predicting various clinical outcomes, ultimately leading to more informed and personalized patient care [44]. For instance, Zang et al. [45] proposed neural networks that combine sequential unstructured clinical notes with structured data for predicting multiple outcomes, including 30-day hospital readmissions. Their fusion models, which utilize document embeddings alongside convolutional neural networks (CNN) or long short-term memory (LSTM) networks, demonstrated improved prediction accuracy over baseline models, with an AUROC of 0.674. However, upon closer inspection, the performance of these models in predicting hospital readmissions was less impressive compared to other tasks, such as in-hospital mortality prediction. This highlights the inherent complexity of modeling readmission risk.

Lin et al. [46] employed a Recurrent Neural Network (RNN) architecture with LSTM layers to capture temporal dependencies and fluctuations in patient data. Their LSTM-based model outperformed traditional machine learning and convolutional neural network (CNN) models in predicting ICU readmissions, achieving a sensitivity of 0.742 and an AUROC of 0.791. The model effectively captured high volatility and unstable physiological states—key indicators of readmission risk—and identified key predictive features, including glucose levels, heart rate, body temperature, Glasgow Coma Scale, and oxygen saturation. Similarly, Rajkomar et al. [47] developed a deep learning framework using patients’ complete raw EHR data, including free-text clinical notes, from the University of California, San Francisco (UCSF) and the University of Chicago Medicine (UCM). Their model, trained on data from 2012–2016 (UCSF) and 2009–2016 (UCM), predicted multiple outcomes, including 30-day unplanned readmissions, with an AUROC of 0.75–0.76.

Johnson et al. [48] investigated the use of unstructured clinical notes for predictive modelling using deep learning architectures, including RNNs, attention-based time-aware neural networks (TANNs), and boosted time-based decision stumps. Their approach demonstrated how incorporating clinical text can enhance model explainability by highlighting note features relevant to predictions. However, the method had limitations in transferability and required substantial computational resources.

A study conducted in Alberta, Canada [49] compared the performance of the traditional LACE model with a Gradient Boosting Machine (GBM) model that incorporated both manually derived features from population-level linked administrative hospital data and machine-learned features. These features included longitudinal patient health records encoded using Word2Vec, a NLP technique. The target outcome was all-cause 30-day hospital readmission. The GBM model significantly outperformed the LACE model, achieving an AUC of 0.83 compared to 0.66.

### Summary of literature review

The literature reveals a clear transition from traditional statistical models to advanced machine learning (ML) and natural language processing (NLP) techniques for hospital readmission prediction. Transformer-based large language models (LLMs), such as BERT, have demonstrated improved predictive performance by effectively leveraging unstructured clinical notes. However, the integration of LLMs with structured data remains underexplored. This study proposes a framework that combines structured electronic health record (EHR) data with unstructured clinical notes, using embeddings from a fine-tuned ClinicalT5 model for both clinical narratives and diagnostic information. This approach aims to investigate the added value of integrating LLM-derived representations with structured features to enhance 30-day hospital readmission prediction. A summary of key studies, including model descriptions, performance metrics, and their respective strengths and limitations, is presented in Table 1.

**Table 1:** Authors, Description & Metric Scores.

Authors	Description	Metric	Pros	Cons
[10]	Uses the LACE index developed by the authors to quantify readmission risk	C-statistic: 0.684	Simple and easy to interpret	Low/Moderate predictive ability
[11]	Develops the HOSPITAL score for easy readmission prediction	C-statistic: 0.71	Incorporates both clinical and claims data for better feature derivation	Moderate predictive ability
[12]	Develops the Patient at risk of readmission within 30 days (PARR-30) model	C-statistic: 0.70	Simple	Low sensitivity
[20]	Logistic Regression using health insurance claims data	AUROC: 0.60	Proves the lack of predictive power of claims data	Used insurance claims Poor discriminative ability
[19]	Gradient Boosting Decision Trees on claims data	AUROC: 0.653	Gradient boosting improves predictive performance	Data lacked important features necessary for patient population.
[22]	CatBoost for 14-day unplanned readmission	AUROC: 0.9909 Recall: 0.56	High predictive accuracy	Exhibits class imbalance as it has high accuracy but poor recall
[24]	XGBoost optimized with Bayesian techniques	AUROC: 0.92 Recall: 0.40	High predictive performance	Imbalanced model performance due to low recall
[25]	Combines a modified weight-boosting algorithm with a stacking algorithm	Recall: 0.891	Addresses Class Imbalance	High computational demand, complex feature engineering
[26]	Uses RandomForest boosting algorithms for predicting readmission risk	Accuracy: 0.99	High Accuracy	Does not use real hospital data
[27]	Introduced an Optimal Variational Bayesian Logistic Regression (OVBLR) model with a Salient Feature Estimation (SFE) strategy	Accuracy: 0.88	Interpretable	High computational demand when scaling to larger datasets
[29]	Utilizes ANNs on EHR features and social determinants of health	AUROC: 0.78	Incorporates social determinants of health	Poor generalization Lacks interpretability
[30]	Neural ordinary differential equations (ODEs) + RNN, and attention-based models for predicting ICU readmission risk	AUC: 0.739 F1: 0.372	Interpretable	Limited improvement over simpler models High computational cost Low F1 Score
[39]	Applies BERT on clinical notes to introduces ClinicalBERT	AUROC: 0.714	Improved performance over LSTMs and BERT	Moderate predictive performance
[40]	Utilizes SciBERT on clinical notes to predict hospital readmission	AUROC: 0.735	Improves on ClinicalBERT	Insufficient Data
[44]	Multi-modal neural network architecture that combines structured and unstructured datas.	AUROC: 0.674	Improved accuracy compared to baseline methods in the same context	Inadequate features Long running time
[45]	RNN with LSTMs for ICU readmission prediction using time series data and ICD9 embeddings	AUROC: 0.791	Comprehensive feature set	Lacks explainability High computational power
[46]	Deep learning approach using entire raw EHR data (FHIR standard), predicting hospital readmission	AUROC: 0.75–0.76	Scalability Generalizable across multiple centers	Heavy computational requirements Challenges with real-time interpretability
[47]	Combines manually derived features and machine-learned features encoded using Word2vec in a Gradient Boosting model to predict 30-day readmission	AUROC: 0.83	High accuracy	Uses linked administrative data

## Materials and methods

### Proposed framework

**Fig 2:**
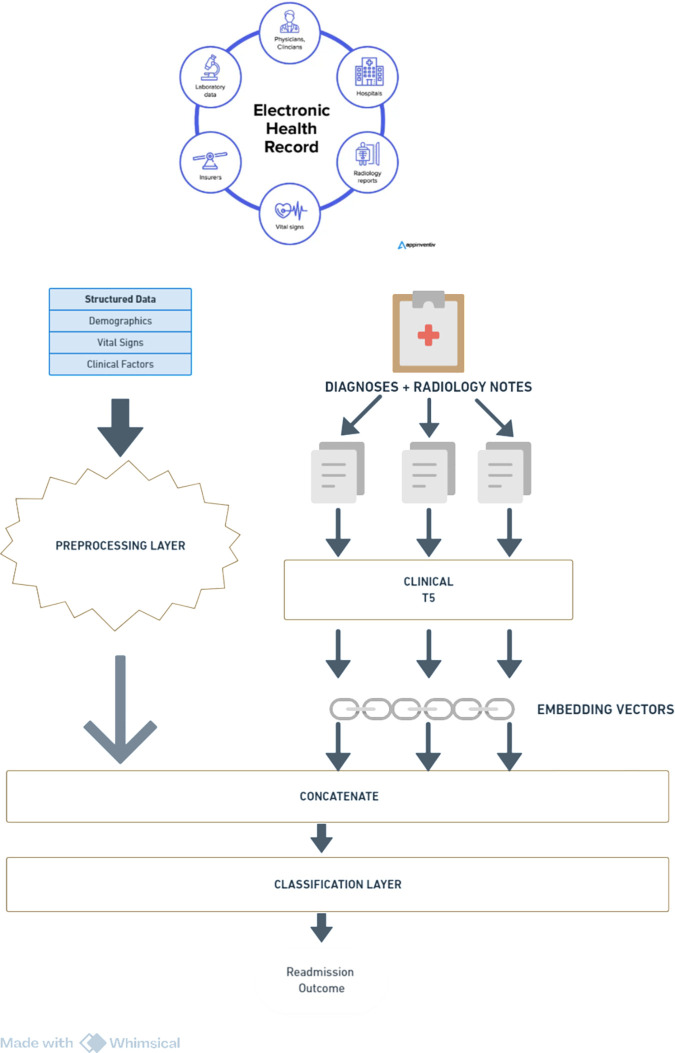
Proposed framework.

We propose a method that constructs a comprehensive patient representation by combining data modalities. The overall framework of the proposed approach is illustrated in Fig 2.

The proposed framework comprises two main components: (1) LLM Embedding Extraction using Clinical-T5, a large language model trained on MIMIC clinical notes [14], and (2) Integration with Structured Data. Text representations are derived from the decoder’s hidden states of Clinical-T5. Structured data undergoes preprocessing, where numerical features are standardized and categorical features are encoded. The resulting embeddings from both modalities are concatenated to form a unified patient representation, which is then input into an ensemble classifier to predict 30-day hospital readmissions.

### LLM embeddings extraction

To perform this task, we selected ClinicalT5, a variant of the T5 model fine-tuned on clinical notes. ClinicalT5 builds upon the original Text-to-Text Transfer Transformer (T5) architecture, an encoder-decoder framework designed for text generation tasks. It retains the core components of the original Transformer model [37], with modifications including changes to the LayerNorm bias, layer normalization, and positional embedding scheme. For detailed architectural insights, readers are referred to the original T5 paper [50].

The version of Clinical T5 used in this study is Clinical-T5-Scratch, which follows the same architecture as T5-Base with 220 million parameters. Clinical-T5-Scratch builds on T5-Base by undergoing additional training on MIMIC-III [32] and MIMIC-IV [51] data with random initialized weights.

To obtain the embeddings, the clinical texts are split into chunks and tokenized to generate input tokens: input_ids, X attention_mask, A and fed to ClinicalT5 in batches. The model processes the input and produces a sequence of hidden states, $\left[h_{1},h_{2},\ldots ,h_{n}\right]$, corresponding to each token.


$$H=T_{5}(X,A)$$

Where,


$$\left[h_{1},h_{2},\ldots ,h_{n}\right]$$

The text representation is obtained by averaging the decoder hidden states through mean pooling across the decoder layers, resulting in a single embedding for the entire input sequence.


$$E=\frac{1}{n}\sum_{i=1}^{n}h_{i}$$

Where,


$$\begin{array}{c} E=\text{Embedding of a single input sequence}\\ n=\text{Number of tokens in the sequence}\\ h_{i}=\text{Hidden state for the }i\text{th token} \end{array}$$

This process was performed in batches of 32 input sequences, with the resulting embeddings stored in a list for future use. These embeddings were then concatenated with pre-processed numerical and categorical data, along with the readmission outcome, to create a comprehensive representation of each patient’s condition during their admission or hospital stay.

### Data source and selection

**Fig 3:**
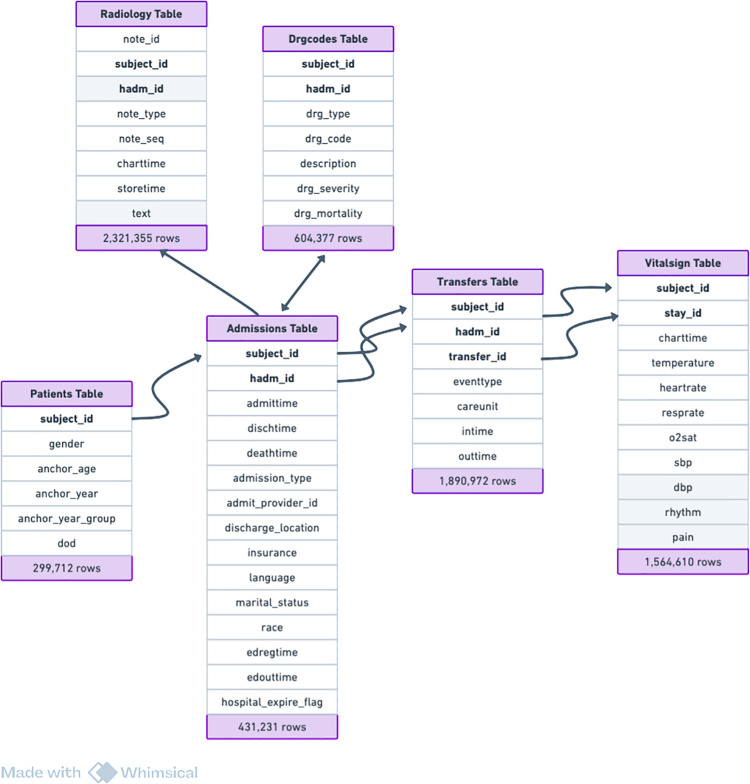
MIMIC-IV database schema and No of records.

**Fig 4:**
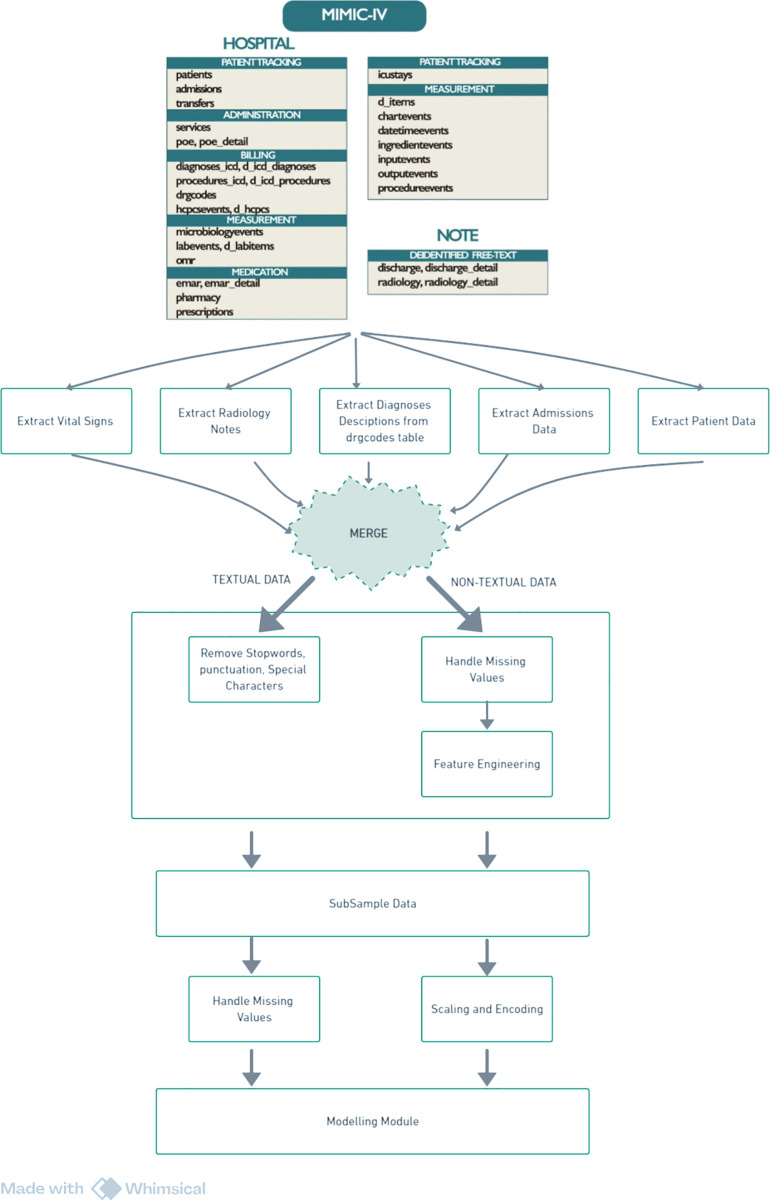
Data pre-processing framework.

This research uses the MIMIC-IV dataset, derived from the electronic health records (EHRs) of Beth Israel Deaconess Medical Center (BIDMC) in Boston, MA, USA [51]. Access was granted through PhysioNet [52] upon completion of CITI training.The dataset includes two primary modules: hosp (our focus) and icu, covering both hospital and ICU stays. The hosp module mainly contains data from hospital admissions, with some from the emergency department. Clinical notes, including 331,794 discharge summaries and 2,321,355 radiology reports, were obtained from MIMIC-IV-Note [51]. For this study, we extracted the admissions, patients, DRG codes, and transfers tables from hosp, the radiology notes table from MIMIC-IV-Note, and the vital signs table from the Emergency module. These tables provide demographic, admission, vital signs, diagnostic, and radiological data essential for our research. The dataset has been de-identified to ensure that no personally identifiable information is included. The dataset was accessed on 04/07/2024. The final dataset integrated the extracted tables into a single comprehensive table, as shown in Fig 3, which represents unique hospital stays. Prior to analysis, the data underwent a thorough cleaning and transformation process Fig 4 to ensure its quality and appropriateness for the study.

### Handling missing value

The missing values in the dataset were addressed on a case-by-case basis using a function, as illustrated in the following Table 2.

**Table 2:** Handling missing value.

Features	Action Taken
language	Rows with “?” replaced with “ENGLISH”
marital status	Imputed with the most frequent value
discharge location	Imputed missing values with “Unknown”
diagnoses	Rows with missing diagnoses description were dropped
drg severity	Null values replaced with 0
drg mortality	Null values replaced with 0
temperature	Column dropped due to a large number of null values
pain	Column dropped due to inconsistent values

### Feature engineering

To enhance the quality of the dataset, several feature engineering procedures were undertaken. A new feature, termed duration, was created to quantify the length of patient hospitalization. The resulting values were rounded up to whole days, as length of stay is usually calculated in whole days. The admission type feature contained multiple synonyms. For example, “EW EMER.”, “URGENT”, and “DIRECT EMER.” can all be represented as “Emergency”. Furthermore, the race column had 33 values which were consolidated into 7 main values: “BLACK”, “WHITE”, “ASIAN”, “HISPANIC”, “HAWAIIAN”, “AMERINDIAN” and “OTHER” for unspecified races. Additionally, data types were appropriately adjusted for the numerical columns from object to “int” or “float” where relevant. These refinements were implemented to improve data uniformity and facilitate more robust subsequent analyses.

### Dropping features

1. The following columns, which did not provide any further benefit for the readmission task, were dropped:
  - Admittime
  - Dischtime
  - edregtime
  - edouttime
  - Next admittime
  - subject id
  - admittime c
2. The columns storing “Next admission type” and “Days until next admission” were dropped, as they are strong predictors of a future readmission, which could bias the model’s performance.

### Data subsampling

**Fig 5:**
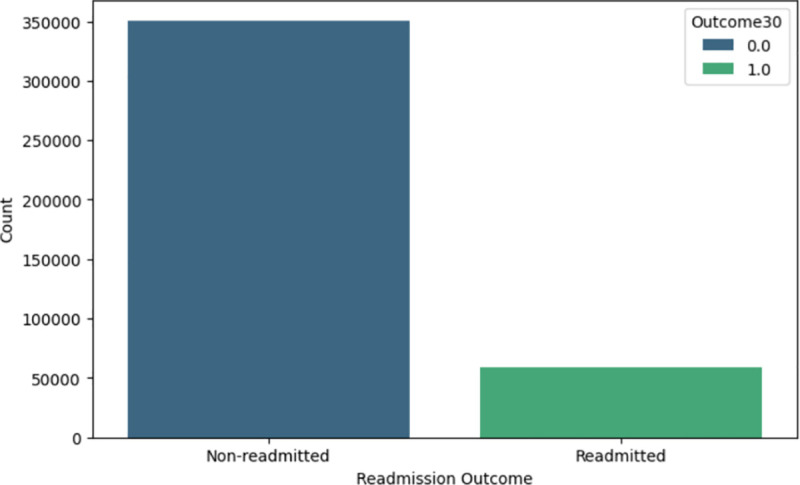
Imbalance datasets (counts of patients).

**Fig 6:**
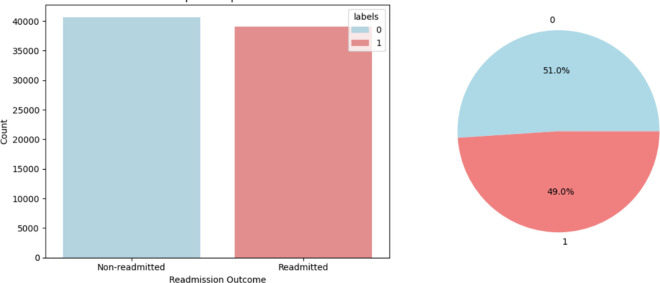
Balance dataset and distribution of readmission outcome.

The dataset exhibited class imbalance, with 268,105 instances of the negative class (class “0”) and 48,743 instances of the positive class (class “1”), as shown in Fig 5. This imbalance could lead to a biased model that favours the negative class. To address this, the negative class was undersampled to match the size of the positive class, resulting in a more balanced dataset, as shown in Fig 6. Undersampling was selected for its simplicity and to prevent the generation of artificial data.

### Feature analysis

The subsampled training set contains 79,731 rows and 21 features, including 10 numerical, 8 categorical, and 2 textual features.

#### Numerical features observations.

**Fig 7:**
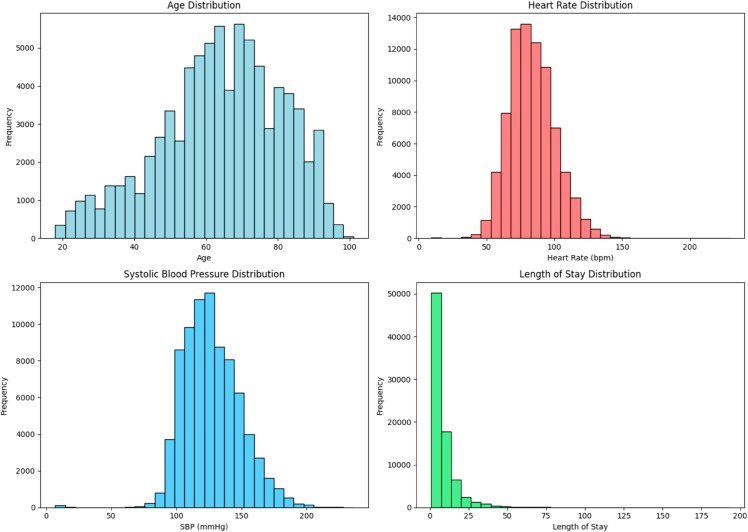
Distribution of numerical features.

The distribution of numerical features is presented in Fig 7.

- **Age:** The distribution is approximately normal, with a peak around 60–70 years, indicating an older population.
- **Heart Rate:** The distribution is close to normal, with most heart rates ranging from 70 to 100 bpm, which is typical for adults.
- **Systolic Blood Pressure:** The distribution centers around 120–150 mmHg, which falls within the normal adult range.
- **Length of Stay:** The distribution is strongly right-skewed, indicating that most patients had relatively short stays.

#### Categorical features.

**Fig 8:**
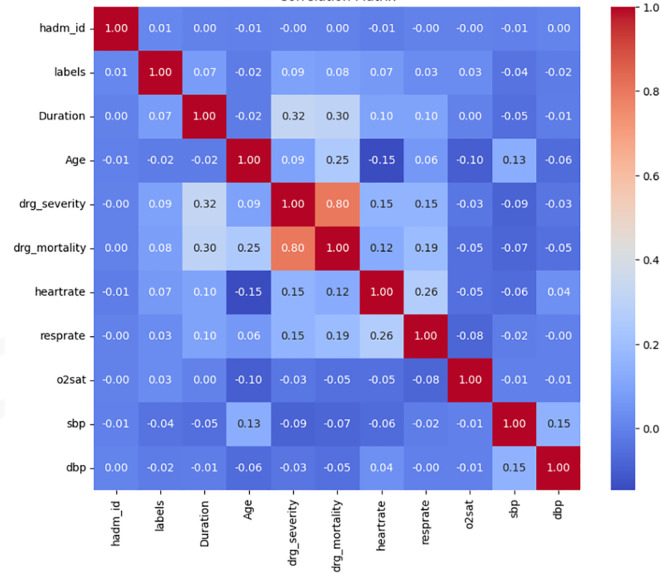
Correlation matrix of features.

**Fig 9:**
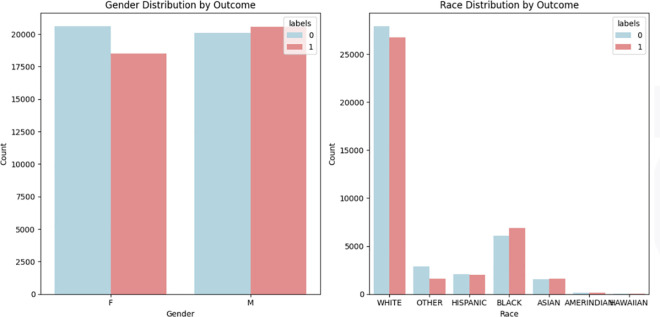
Distribution of demographic features.

**Fig 10:**
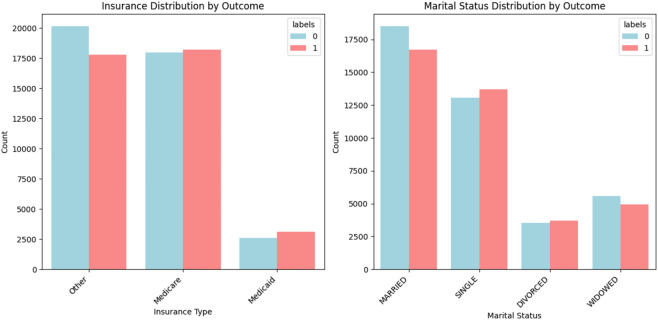
Distribution of socio economic features.

The distribution of categorical features is shown in Fig 8 (correlation matrix), Fig 9 (demographics), and Fig 10 (socioeconomic features). The key observations for these categorical features are as follows:

- **Admission Location:** Patients admitted from the emergency room are more likely to have another emergency readmission.
- **Gender:** Slightly more females are associated with negative outcomes, while slightly more males are linked to positive outcomes.
- **Race:** The dataset predominantly consists of white individuals, with a balanced distribution across outcomes but a slightly higher count in the negative class. Black individuals show a balanced distribution, and other races have smaller counts with a fairly balanced distribution.
- **Insurance Type:** The ’Other’ insurance type has a higher count in the negative class. Medicare has an almost even distribution across both classes, while Medicaid has a smaller overall count, with a higher proportion in the positive class.
- **Marital Status:** More married individuals are associated with the negative outcome.

#### Textual data.

**Fig 11:**
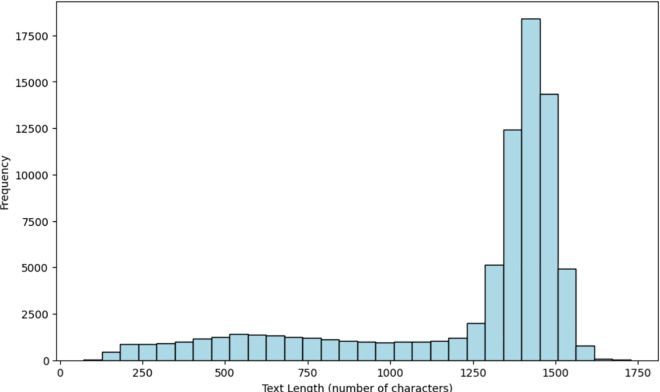
Combined text length distribution.

The text length distribution is left-skewed, with most entries ranging from 1300 to 1500 characters, as shown in Fig 11.

## Model development

In this study, two primary experiments were conducted to evaluate the efficacy of the proposed approach::

### Proposed method

Hospital readmission prediction was performed using tabular data and text embeddings extracted via ClinicalT5.

#### Tabular data + ClinicalT5 text embedding + classifier.

In this study, we developed a model that combines structured patient data with unstructured clinical notes. We fine-tuned ClinicalT5 for 5 epochs using the Hugging Face Trainer API [62]. Further training was not conducted due to computational constraints. The training parameters for ClinicalT5 are provided in the Table 3.

**Table 3:** ClinicalT5 parameters.

Parameter	Value
Learning rate	3e-5
Epochs	5
Batch size	16
Optimizer	Adam W

#### Neural network.

We constructed a feed-forward neural network comprising an input layer, three hidden layers, and an output layer. The network’s architecture and parameters are shown in Table 4 and Table 5, respectively. The training process was managed by a *train_model* method, which oversees the entire training pipeline.

**Initialization**: The model is set to training mode using *self.train()*. A history dictionary is initialized to store loss values, and variables for early stopping are prepared.

**Epoch Loop**: The model is trained for a specified number of epochs, iterating over the dataset multiple times.

- For each epoch, the training dataset is divided into batches. For each batch:
  – A forward pass is performed, where the input data is passed through the model to generate predictions.
  – The loss is computed by comparing the predictions to the true labels using a loss function (criterion).
  – The gradients of the loss with respect to the model’s weights are calculated via backpropagation (*loss.backward()*), and the optimizer updates the model’s weights accordingly
- The average training loss across all batches is recorded at the end of each epoch.

**Validation:** After each epoch, the model is evaluated on the validation dataset, and the corresponding loss is computed.

**Table 4:** Neural network model description.

Layer	Descriptions	Parameters
Input Layer	Fully Connected (fc1)	Input Dim: 806, Hidden Dim: 512
Activation (1)	ReLU	N/A
Dropout (1)	Dropout Layer	Dropout Rate: 0.25
Hidden Layer (2)	Fully Connected (fc2)	Hidden Dim: 256
Activation (2)	ReLU	N/A
Dropout (2)	Dropout Layer	Dropout Rate: 0.25
Hidden Layer (3)	Fully Connected (fc3)	Hidden Dim: 64
Activation (3)	ReLU	N/A
Output Layer	Fully Connected (fc4)	Output Dim: 1
Activation (4)	Sigmoid	N/A

**Table 5:** Model training parameters.

Parameter	Value
Learning Rate	3*e*^−5^
Epochs	10
Optimizer	AdamW
Weight Decay	1*e*^−4^
Loss Function	Binary Cross Entropy Loss

**Comparison method:** PubMedBERT, a Large Language Model (LLM), was fine-tuned exclusively on free-text data for the task of predicting hospital readmission, as detailed in the Comparison Method section.

### Comparison method

In this study, we fine-tuned the PubMedBERT transformer model [53] on the dataset’s text and diagnosis description variables to enable a comparative analysis. The dataset used in this experiment differs slightly from others, primarily due to its larger number of text entries, which allowed for more extensive model training. However, the dataset underwent pre-processing and subsampling following the same procedures described in previous sections. The specific variant of PubMedBERT used in this experiment is the PubMedBERTMNLI-MedNLI model, introduced by [54]. This model was initially fine-tuned on the Multi-Genre Natural Language Inference (MNLI) dataset [55], and subsequently further fine-tuned on the MedNLI dataset [56].

#### Fine-tuning.

The model was fine-tuned for the hospital readmission task using the radiology notes and diagnosis texts. Various hyperparameters were explored, and we ultimately selected a learning rate of 5*e*^−5^, a batch size of 32, and 5 epochs. Validation was performed every 3,000 steps, with results recorded and model checkpoints saved. At the conclusion of training, the best model was chosen based on the lowest training loss.

The training parameters for PubMedBERT are presented in Table 6.

**Table 6:** PubMedBERT training parameters.

Parameter	Value
Learning Rate	5*e*^−5^
Epochs	5
Batch Size	32
Optimizer	AdamW

## Experimental setup

The experiments were conducted on a cloud computing platform equipped with 16-core, 48GB NVIDIA L40 GPUs. Tools such as sklearn [57] were used to compute evaluation metrics and import the the necessary modelling libraries, while PyTorch was used to construct the Neural Network Classifier [58].

### Evaluation matrix

To assess the performance of the models across the different experiments, the following evaluation metrics were employed:

**Accuracy:** is defined as the proportion of correctly predicted outcomes relative to the total number of predictions, and is calculated as follows:


$$\text{Accuracy}=\frac{\text{Number of Correct Predictions}}{\text{Total Number of Predictions}}\times 100$$

**Recall:** Also referred to as sensitivity or the true positive rate, recall assesses the model’s ability to correctly identify actual positive cases. It is calculated as:


$$\text{Recall}=\frac{\text{True Positives}}{\text{True Positives}+\text{False Negatives}}$$

**Precision:** This metric evaluates how accurately the model predicts the positive class among all instances classified as positive. It focuses on minimizing false positives. Precision is calculated as:


$$\text{Precision}=\frac{\text{True Positives}}{\text{True Positives}+\text{False Negatives}}$$

**Specificity:** Also referred to as the true negative rate, specificity measures how effectively the model identifies negative cases. It is calculated as:


$$\text{Specificity}=\frac{\text{True Negatives}}{\text{True Negatives}+\text{False Positives}}$$

**Area Under the Receiver Operating Characteristic Curve (AUROC):** The AUROC curve reflects the likelihood that a randomly chosen positive instance is ranked higher than a negative one. It provides an aggregate measure of a model’s ability to distinguish between positive and negative classes across all classification thresholds. A higher AUROC indicates better model performance [59].

**F1 Score:** The F1 Score is the harmonic mean of Precision and Recall, offering a balanced measure between the two metrics. It is especially useful in cases with imbalanced class distributions, where both false positives and false negatives are significant. The F1 Score is calculated as:


$$F1\;Score=\frac{2\times \text{Precision}\times \text{Recall}}{\text{Precision}+\text{Recall}}$$

**Matthews Correlation Coefficient (MCC):** The MCC is a correlation coefficient between actual and predicted binary classifications, considering true positives, true negatives, false positives, and false negatives. It is a balanced metric, particularly useful for imbalanced datasets, as it offers a more informative measure of model performance than accuracy. The MCC is calculated as: [60].


$$\text{MCC}=\frac{TP\times TN-FP\times FN}{\sqrt{\left(TP+FP)(TP+FN)(TN+FP)(TN+FN\right)}}$$

## Results

In this study, we fine-tuned the PubMedBERT transformer model [53] on the dataset’s text and diagnosis descriptions for comparison. The dataset, containing more text entries than others, was pre-processed and subsampled as described in earlier sections. We employed the PubMedBERTMNLI-MedNLI variant [54], initially fine-tuned on the Multi-Genre Natural Language Inference (MNLI) dataset [55] and further refined on the MedNLI dataset [56].

### PubMedBERT with text only

**Fig 12:**
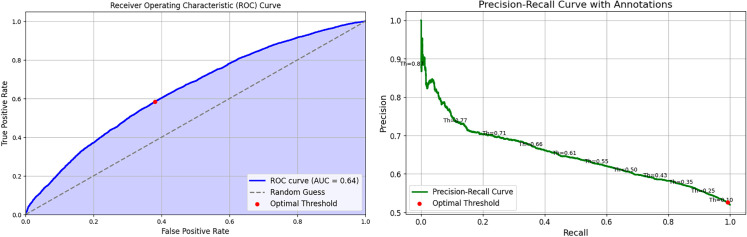
PubMedBERT AUROC Curve (left) and PubMedBERT Precision-Recall Curve (right).

The results of applying PubMedBERT to the clinical notes, including the AUROC curve (left) and Precision-Recall curve (right), are shown in Fig 12. With an AUROC score of 0.64, the model performs only marginally better than random chance (0.5). As seen in Fig 12 (left), the AUROC curve demonstrates the model’s performance in comparison to random guessing. Although the results are moderate, there is significant potential for improvement.

**Fig 13:**
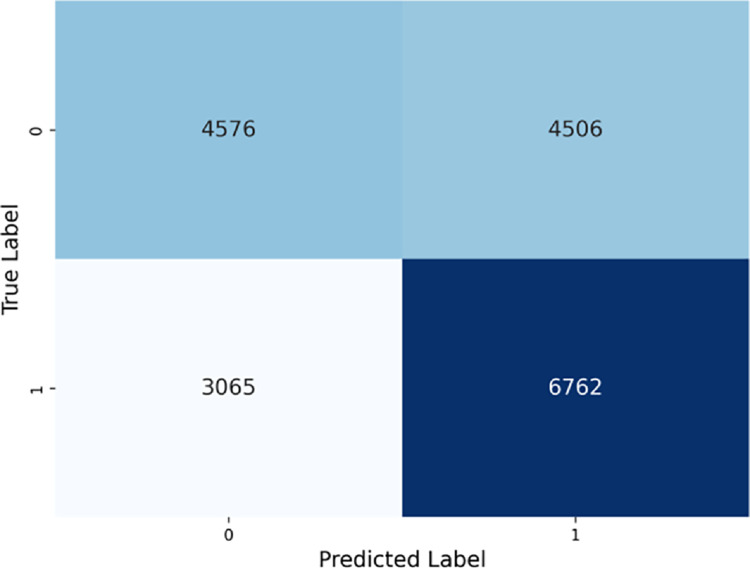
PubMedBERT confusion matrix.

The confusion matrix in Fig 13 visually confirms the model’s imbalanced performance, showing a high number of true positives, consistent with the relatively high recall observed.

#### ClinicalT5 + tabular data with classifiers.

The results of the four classifiers: Neural Network, Extreme Gradient Boosting (XGBoost) Classifier, Light Gradient Boosting Machine (LGBM) Classifier, and Voting Classifier—applied to the ClinicalT5 embeddings combined with the processed structured data are presented below.

#### Neural network.

**Fig 14:**
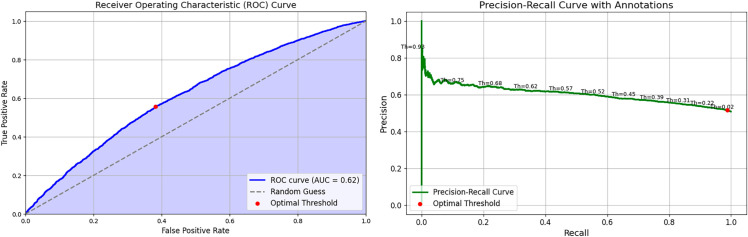
Neural network performance evaluation curves.

The Neural Network’s AUROC score, shown in Fig 14 (left) demonstrates moderate discriminative ability, slightly outperforming PubMedBERT. Similarly, Fig 14 (right) visualizes the trade-off between precision and recall at various thresholds.

**Fig 15:**
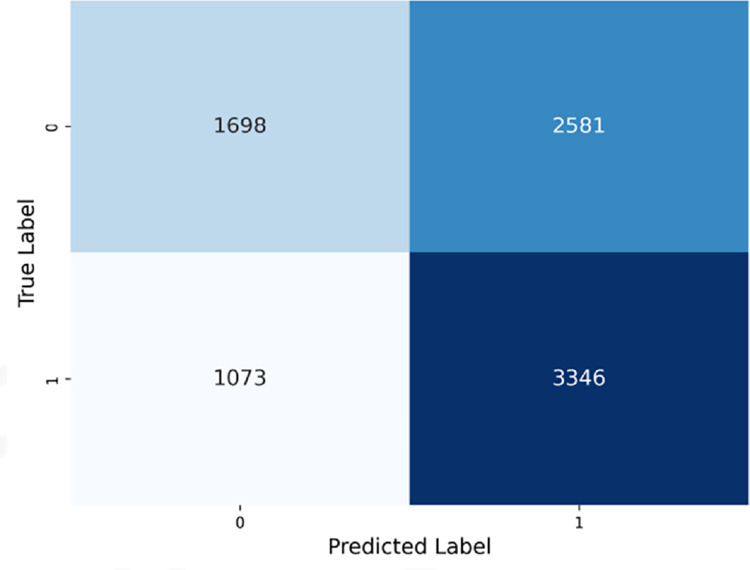
Neural network confusion matrix.

The confusion matrix in Fig 15 confirms the model’s imbalanced performance, with a high number of true positives and false positives.

#### Extreme Gradient Boosting (XGBoost) classifier.

**Fig 16:**
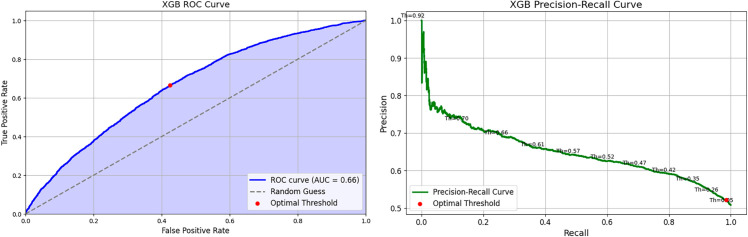
Extreme gradient boosting classifier performance evaluation curves.

The performance of the XGBoost Classifier is shown in Fig 16, with the AUROC curve (left) and Precision-Recall curve (right). This model demonstrates improved accuracy compared to the Neural Network Classifier and PubMedBERT. The recall and precision are closely aligned, indicating a more balanced approach to predicting both positive and negative cases.

**Fig 17:**
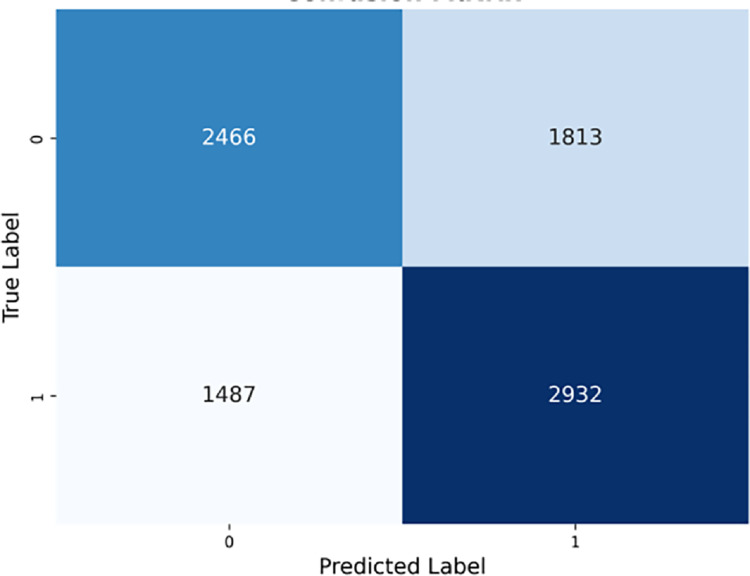
Extreme gradient boosting classifier confusion matrix.

The confusion matrix in Fig 17 shows an improved balance across the quadrants, with the model successfully identifying more true negatives and true positives.

#### Light Gradient Boosting (LGBM) classifier.

**Fig 18:**
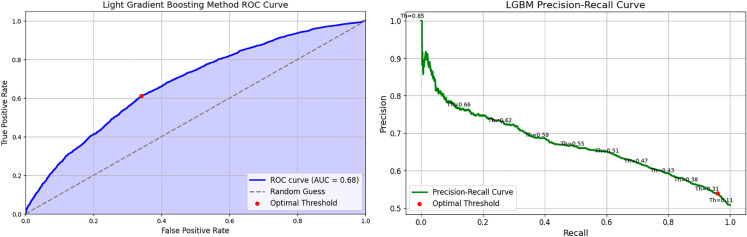
Light gradient boosting classifier performance evaluation curves.

The performance of the LGBM Classifier is shown in Fig 18, with the AUROC curve (left) and Precision-Recall curve (right). The AUROC curve in Fig 18 (left) shifts more to the left, indicating a better balance between sensitivity and specificity. The precision-recall curve in Fig 18 (right) demonstrates a higher optimal threshold for the precision-recall trade-off.

**Fig 19:**
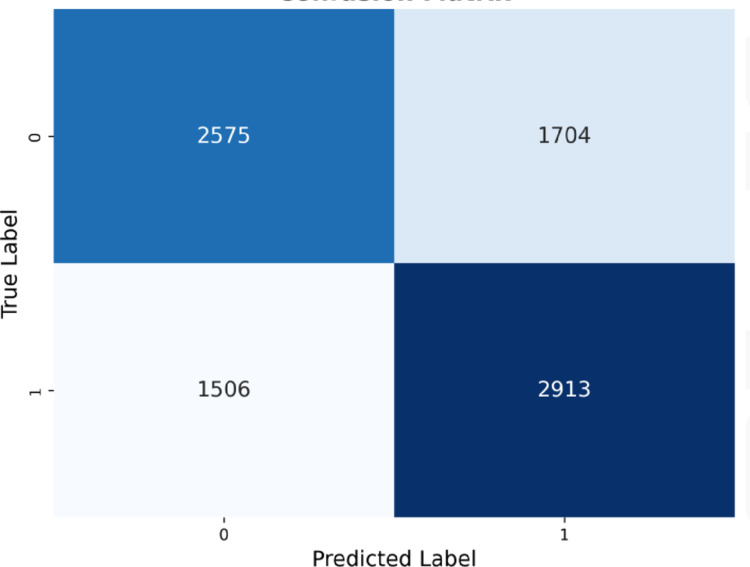
Light gradient boosting confusion matrix.

The confusion matrix in Fig 19 shows a more balanced distribution across the quadrants, with the model successfully identifying a greater number of true negatives and true positives.

#### Voting classifier.

**Fig 20:**
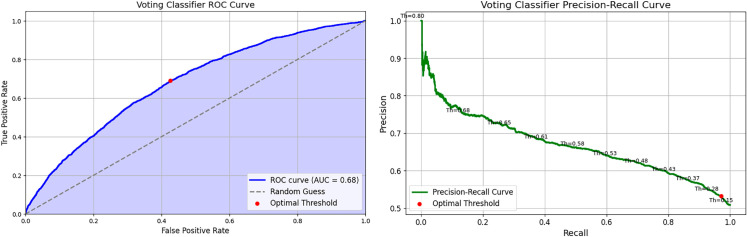
Voting classifier performance evaluation curves.

The performance of the Voting Classifier is shown in Fig 20, with the AUROC curve (left) and Precision-Recall curve (right). The AUROC curve in Fig 20 (left) indicates a better optimal threshold, with an AUROC score of 0.68, matching the LGBM Classifier and reflecting a moderate ability to distinguish between classes. Fig 20 (right) shows a lower optimal threshold for the precision-recall trade-off when compared to the LGBM Classifier.

**Fig 21:**
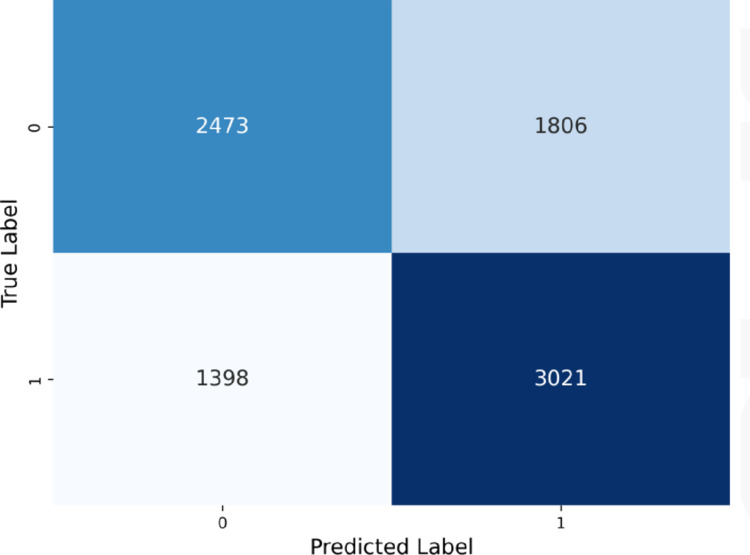
Voting classifier confusion matrix.

The confusion matrix in Fig 21 shows a higher number of true positives, consistent with the high recall recorded, along with an increased number of false positives.

## Comparative results

The performance of the models is evaluated and compared across several metrics, including accuracy, recall, precision, and AUROC scores. The results in Table 7 provide insights into the strengths and limitations of the proposed hybrid model in comparison to text-only models, highlighting areas where the hybrid approach excelled and where it fell short.

**Table 7:** Performance metrics of different models.

Models	Accuracy	Recall	Precision	Specificity	F1 Score	AUROC	MCC
ClinicalT5 + LGBM	0.63	0.66	0.63	0.60	0.64	0.68	0.26
ClinicalT5 + VotingClassifier	0.63	0.68	0.63	0.58	0.65	0.68	0.26
ClinicalT5 + XGBClassifier	0.62	0.66	0.62	0.58	0.64	0.66	0.24
PubMedBERT	0.60	0.69	0.60	0.50	0.64	0.64	0.20
ClinicalT5 + Neural Network	0.58	0.76	0.56	0.40	0.65	0.62	0.17

**Fig 22:**
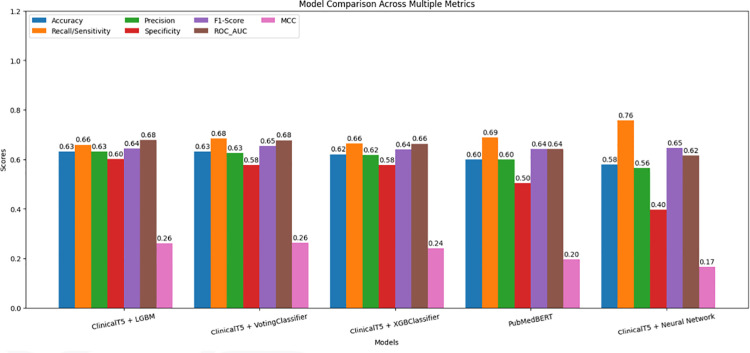
Model comparison across multiple metrics with bar chart.

**Fig 23:**
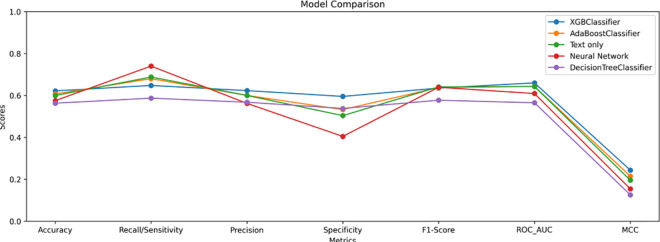
Model comparison across multiple metrics with line chart.

The performance of the models is compared across multiple metrics, as illustrated in the bar chart (Fig 22) and the line chart (Fig 23).

**Fig 24:**
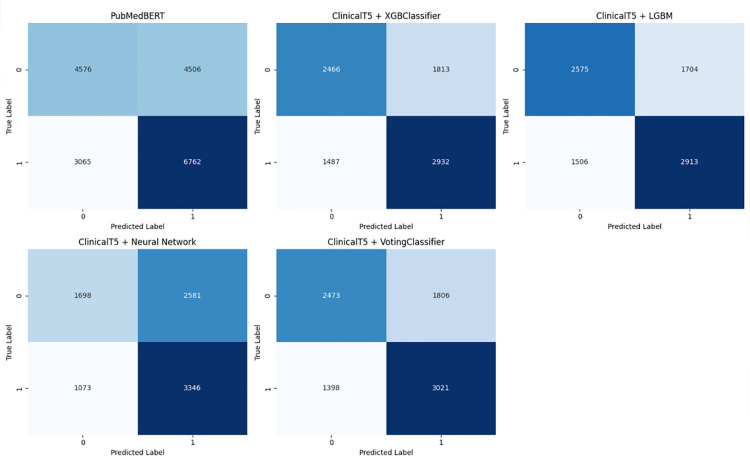
Confusion matrices for all models.

The confusion matrices for all models are shown in Fig 24.

As seen in the confusion matrices, PubMedBERT focuses heavily on true positives, resulting in high recall, but also suffers from a substantial number of false positives. ClinicalT5+NeuralNetwork exhibits a similar pattern.

In contrast, the hybrid ensemble models display a more balanced approach, with relatively fewer false positives and a higher number of true positives. These models demonstrate a lower rate of misclassifications when compared to the other two models.

### To summarize

**PubMedBERT and ClinicalT5+NeuralNetwork** prioritize recall but are affected by a high number of false positives, which leads to lower precision and specificity. Consequently, these models also exhibit the lowest accuracy among the five models considered.

**ClinicalT5+LGBM and ClinicalT5+VotingClassifier** outperform PubMedBERT across most metrics, particularly in terms of precision-recall balance and AUROC. This makes them more reliable for predicting hospital readmissions.

## Result analysis

**Accuracy:** The ClinicalT5+LGBM and ClinicalT5+VotingClassifier models achieved the highest accuracy among all models in this study, both with AUROC scores of 0.68. This indicates their superior performance in distinguishing between positive and negative cases. In comparison, PubMedBERT scored lower at 0.64, suggesting that the hybrid models provide more reliable predictions overall.

**Recall:** The ClinicalT5+NeuralNetwork model achieved the highest recall (0.76), although it was accompanied by lower precision. Similarly, PubMedBERT exhibited a strong recall score of 0.69, with slightly higher precision (0.60). On the other hand, the hybrid ensemble model, ClinicalT5+LGBM, demonstrated a more balanced approach, achieving a recall of 0.66 and precision of 0.63. This indicates that ClinicalT5+LGBM was more effective at managing false positives while maintaining a good recall rate.

**Specificity:** The specificity results revealed that ClinicalT5+NeuralNetwork faced challenges in distinguishing negative cases, recording a specificity score of 0.40. Similarly, PubMedBERT exhibited a comparable performance with a specificity score of 0.50. In contrast, the ClinicalT5+LGBM model demonstrated a notable improvement, achieving a specificity of 0.60. This indicates a stronger capability to accurately identify non-readmission cases, highlighting its superior performance in distinguishing between positive and negative instances.

**MCC:** he Matthews Correlation Coefficient (MCC) scores were generally low across all models. However, ClinicalT5+LGBM and ClinicalT5+VotingClassifier achieved the highest MCC scores of 0.26, indicating that these models are more effective at generalizing their predictions.

## Discussion

The performance of the ClinicalT5 + Ensemble models demonstrates the effectiveness of combining diverse data modalities for predicting hospital readmission. Notably, the ClinicalT5 + LGBMClassifier models outperformed PubMedBERT across several key metrics, including accuracy, precision-recall balance, and AUROC.

These findings emphasize the models’ enhanced capacity to reduce false positives, which is a critical consideration in hospital readmission prediction. In this context, false positives are as problematic as false negatives. Misclassifying a patient as likely to be readmitted could result in unnecessary resource allocation for interventions and treatments. On the other hand, incorrectly classifying a patient as not needing readmission might lead to premature discharge, putting the patient at risk. Therefore, it is crucial to develop models that achieve a balanced trade-off between specificity, precision, and recall.

### Comparison with other studies

The results of this study are consistent with broader trends in hospital readmission prediction research, where models that incorporate structured data tend to outperform those that rely exclusively on unstructured text. For instance, studies by [45] and [47] demonstrated that integrating electronic health record (EHR) data with clinical notes significantly enhances model performance in predicting patient outcomes, including readmissions. Notably, the performance of our model exceeds that of the study by [45].

### Key differences and improvements

Text Segmentation: In this study, clinical notes were processed using ClinicalT5 in manageable chunks of 200 words, allowing the model to better retain the richness of the clinical text without being overwhelmed.

LLM Usage: Unlike the approach taken by [47], which utilized recurrent models that often struggle with long clinical notes, this study integrated structured data with a Large Language Model (LLM). While this method offers improved performance, it comes with a trade-off: LLMs are computationally intensive, which may limit their practicality in smaller healthcare settings with limited resources.

### Strengths and drawbacks

#### Strengths.

**Data integration:** Integrating text representations derived from ClinicalT5 with structured data proved effective in improving predictive performance. Although the overall accuracy remains modest and warrants further enhancement, the proposed hybrid model outperformed the model trained exclusively on unstructured text.

**Balanced results:** The hybrid model exhibited a more balanced performance compared to the text-only model. It successfully identified a substantial proportion of positive cases while reducing the number of false positives.

#### Drawbacks.

**Computational limitations:** One notable limitation of this study was the inability to fine-tune ClinicalT5 beyond five epochs due to constrained computational resources. As a result, training was capped at five epochs before extracting decoder embeddings for use as text representations. This limitation is common in healthcare AI research and has been documented in previous studies [26,47].

**Data quality:** The structured data extracted from the MIMIC-IV database, such as demographics and vital signs, showed a weak correlation with hospital readmission outcomes, which likely contributed to the model’s relatively low overall performance. Hospital readmission prediction is inherently complex and requires consideration of a wide array of factors, including social determinants of health [61], many of which were not captured in the available dataset.

## Conclusion

Hospital readmission remains a major challenge for healthcare systems globally, given its financial burden and implications for patient outcomes. While traditional predictive models offer interpretability and have proven useful, they often fail to capture the full complexity of a patient’s clinical profile, particularly the nuanced information embedded within unstructured clinical notes.

This study sought to address that limitation by investigating whether the integration of unstructured text representations—derived from ClinicalT5—with structured data could enhance prediction performance compared to models relying solely on text. PubMedBERT served as a benchmark model to assess the relative effectiveness of this hybrid approach. The findings indicate that combining structured and unstructured data leads to improved model performance across several key metrics, supporting the potential of multimodal learning in clinical predictive tasks such as hospital readmission.

The research addressed the following questions:

**Research Question 1:** How does integrating 200-word text segments, processed using ClinicalT5, with structured healthcare data, enhance hospital readmission predictions?

The results of this study clearly indicate that the hybrid model, ClinicalT5 + LGBMClassifier, outperforms the text-only model, PubMedBERT, across all evaluation metrics. This finding suggests that the combination of structured healthcare data and clinical note representations can significantly improve predictive accuracy. Moreover, it highlights the potential for further enhancement through additional fine-tuning of the ClinicalT5 model. By integrating diverse data modalities, the hybrid model offers a more comprehensive view of patient conditions, thereby strengthening the prediction of hospital readmissions.

**Research Question 2:** How does this segmented approach compare in terms of prediction accuracy and scalability to traditional methods that use truncated clinical texts?

While the proposed method outperformed PubMedBERT, it did not lead to a substantial improvement in prediction accuracy. This suggests that the choice of segmentation may not have provided a significant advantage in this particular case. Additionally, the high computational resource requirements for fine-tuning and deploying transformer-based models like ClinicalT5 present a notable challenge, especially regarding scalability and their practical applicability in real-world healthcare settings.

## Supporting information

S1 - Guidelines Accessing Dataset This guideline provides instructions for accessing the Medical Information Mart for Intensive Care IV (MIMIC-IV) dataset. (DOCX)

## References

[1] Jencks SF, Williams MV, Coleman EA. Rehospitalizations among patients in the Medicare fee-for-service program. N Engl J Med. 2009;360(14):1418–28. doi: 10.1056/NEJMsa0803563 19339721

[2] Emergency Readmissions. Nuffield Trust. Abstract: This indicator looks at patients who are readmitted to hospital in an emergency within 30 days of discharge. [cited 2025 May 10]. https://www.nuffieldtrust.org.uk/resource/emergency-readmissions

[3] Friebel R, Hauck K, Aylin P, Steventon A. National trends in emergency readmission rates: a longitudinal analysis of administrative data for England between 2006 and 2016. BMJ Open. 2018;8(3):e020325. doi: 10.1136/bmjopen-2017-020325 29530912 PMC5857687

[4] Wadhera RK, Yeh RW, Joynt Maddox KE. The hospital readmissions reduction program - time for a reboot. N Engl J Med. 2019;380(24):2289–91. doi: 10.1056/NEJMp1901225 31091367 PMC6589834

[5] Zhang D, Gurvich I, Van Mieghem J, Park E, Young R, Williams M. Hospital readmissions reduction program: an economic and operational analysis. Management Science. 2016;62:3351–71.

[6] Leppin AL, Gionfriddo MR, Kessler M, Brito JP, Mair FS, Gallacher K, et al. Preventing 30-day hospital readmissions: a systematic review and meta-analysis of randomized trials. JAMA Intern Med. 2014;174(7):1095–107. doi: 10.1001/jamainternmed.2014.1608 24820131 PMC4249925

[7] Department of Health and Social Care. Hospital discharge and community support guidance. 2024. GOV.UK. https://www.gov.uk/government/publications/hospital-discharge-and-community-support-guidance/hospital-discharge-and-community-support-guidance

[8] Bailey MK, Weiss AJ, Barrett ML, Jiang HJ. Characteristics of 30-day all-cause hospital readmissions 2010 –2016. 248. Rockville (MD): Agency for Healthcare Research and Quality (US); 2019. https://www.hcup-us.ahrq.gov/reports/statbriefs/sb248-Hospital-Readmissions.pdf 30896914

[9] Kansagara D, Chiovaro JC, Kagen D, Jencks S, Rhyne K, O’Neil M. Transitions of care from hospital to home: an overview of systematic reviews and recommendations for improving transitional care in the veterans health administration. Washington (DC): Department of Veterans Affairs (US); 2015. 26312362

[10] van Walraven C, Dhalla IA, Bell C, Etchells E, Stiell IG, Zarnke K, et al. Derivation and validation of an index to predict early death or unplanned readmission after discharge from hospital to the community. CMAJ. 2010;182(6):551–7. doi: 10.1503/cmaj.091117 20194559 PMC2845681

[11] Donzé J, Aujesky D, Williams D, Schnipper JL. Potentially avoidable 30-day hospital readmissions in medical patients: derivation and validation of a prediction model. JAMA Intern Med. 2013;173(8):632–8. doi: 10.1001/jamainternmed.2013.3023 23529115

[12] Billings J, Blunt I, Steventon A, Georghiou T, Lewis G, Bardsley M. Development of a predictive model to identify inpatients at risk of re-admission within 30 days of discharge (PARR-30). BMJ Open. 2012;2(4):e001667. doi: 10.1136/bmjopen-2012-001667 22885591 PMC3425907

[13] Salehinejad H, Sankar S, Barfett J, Colak E, Valaee S. Recent advances in recurrent neural networks. arXiv preprint 2018. https://arxiv.org/abs/1801.01078v3

[14] Lehman E, Johnson A. Clinical-T5: large language models built using MIMIC clinical text. 2023. https://www.physionet.org/content/clinical-t5/1.0.0/

[15] Zhao P, Yoo I. A systematic review of highly generalizable risk factors for unplanned 30-day all-cause hospital readmissions. J Health Med Informatics. 2017;8(04).

[16] Talwar A, Lopez-Olivo MA, Huang Y, Ying L, Aparasu RR. Performance of advanced machine learning algorithms overlogistic regression in predicting hospital readmissions: a meta-analysis. Explor Res Clin Soc Pharm. 2023;11:100317. doi: 10.1016/j.rcsop.2023.100317 37662697 PMC10474076

[17] Rajaguru V, Han W, Kim TH, Shin J, Lee SG. LACE index to predict the high risk of 30-day readmission: a systematic review and meta-analysis. J Pers Med. 2022;12(4):545. doi: 10.3390/jpm12040545 35455661 PMC9024499

[18] Artetxe A, Beristain A, Graña M. Predictive models for hospital readmission risk: a systematic review of methods. Comput Methods Programs Biomed. 2018;164:49–64. doi: 10.1016/j.cmpb.2018.06.006 30195431

[19] Zhou H, Della PR, Roberts P, Goh L, Dhaliwal SS. Utility of models to predict 28-day or 30-day unplanned hospital readmissions: an updated systematic review. BMJ Open. 2016;6(6):e011060. doi: 10.1136/bmjopen-2016-011060 27354072 PMC4932323

[20] Min X, Yu B, Wang F. Predictive modeling of the hospital readmission risk from patients’ claims data using machine learning: a case study on COPD. Sci Rep. 2019;9(1):2362. doi: 10.1038/s41598-019-39071-y 30787351 PMC6382784

[21] Brüngger B, Blozik E. Hospital readmission risk prediction based on claims data available at admission: a pilot study in Switzerland. BMJ Open. 2019;9(6):e028409. doi: 10.1136/bmjopen-2018-028409 31256033 PMC6609042

[22] Mahmoudi E, Kamdar N, Kim N, Gonzales G, Singh K, Waljee AK. Use of electronic medical records in development and validation of risk prediction models of hospital readmission: systematic review. BMJ. 2020;369:m958. doi: 10.1136/bmj.m958 32269037 PMC7249246

[23] Raj Pandey S, Ma J, Lai C-H, Raj Regmi P. A supervised machine learning approach to generate the auto rule for clinical decision support system. Trends Med. 2020;20(3). doi: 10.15761/tim.1000232

[24] Lo Y-T, Liao JC, Chen M-H, Chang C-M, Li C-T. Predictive modeling for 14-day unplanned hospital readmission risk by using machine learning algorithms. BMC Med Inform Decis Mak. 2021;21(1):288. doi: 10.1186/s12911-021-01639-y 34670553 PMC8527795

[25] González-Nóvoa JA, Campanioni S, Busto L, Fariña J, Rodríguez-Andina JJ, Vila D, et al. Improving intensive care unit early readmission prediction using optimized and explainable machine learning. Int J Environ Res Public Health. 2023;20(4):3455. doi: 10.3390/ijerph20043455 36834150 PMC9960143

[26] Yu K, Xie X. Predicting hospital readmission: a joint ensemble-learning model. IEEE J Biomed Health Inform. 2020;24(2):447–56. doi: 10.1109/JBHI.2019.2938995 31484143

[27] Lee H, Kim S, Moon H-W, Lee H-Y, Kim K, Jung SY, et al. Hospital length of stay prediction for planned admissions using observational medical outcomes partnership common data model: retrospective study. J Med Internet Res. 2024;26:e59260. doi: 10.2196/59260 39576284 PMC11624451

[28] Liu J, Wu X, Xie Y, Tang Z, Xie Y, Gong S. Small samples-oriented intrinsically explainable machine learning using Variational Bayesian Logistic Regression: an intensive care unit readmission prediction case for liver transplantation patients. Expert Systems with Applications. 2024;235:121138. doi: 10.1016/j.eswa.2023.121138

[29] Teo K, Yong CW, Chuah JH, Hum YC, Tee YK, Xia K, et al. Current trends in readmission prediction: an overview of approaches. Arab J Sci Eng. 2021:1–18. doi: 10.1007/s13369-021-06040-5 34422543 PMC8366485

[30] Jamei M, Nisnevich A, Wetchler E, Sudat S, Liu E. Predicting all-cause risk of 30-day hospital readmission using artificial neural networks. PLoS One. 2017;12(7):e0181173. doi: 10.1371/journal.pone.0181173 28708848 PMC5510858

[31] Barbieri S, Kemp J, Perez-Concha O, Kotwal S, Gallagher M, Ritchie A, et al. Benchmarking deep learning architectures for predicting readmission to the ICU and describing patients-at-risk. Sci Rep. 2020;10(1):1111. doi: 10.1038/s41598-020-58053-z 31980704 PMC6981230

[32] Johnson AEW, Pollard TJ, Shen L, Lehman L-WH, Feng M, Ghassemi M, et al. MIMIC-III, a freely accessible critical care database. Sci Data. 2016;3:160035. doi: 10.1038/sdata.2016.35 27219127 PMC4878278

[33] Sheikhalishahi S, Miotto R, Dudley JT, Lavelli A, Rinaldi F, Osmani V. Natural language processing of clinical notes on chronic diseases: systematic review. JMIR Med Inform. 2019;7(2):e12239. doi: 10.2196/12239 31066697 PMC6528438

[34] Wu S, Roberts K, Datta S, Du J, Ji Z, Si Y, et al. Deep learning in clinical natural language processing: a methodical review. J Am Med Inform Assoc. 2020;27(3):457–70. doi: 10.1093/jamia/ocz200 31794016 PMC7025365

[35] Christodoulou E, Ma J, Collins GS, Steyerberg EW, Verbakel JY, Van Calster B. A systematic review shows no performance benefit of machine learning over logistic regression for clinical prediction models. J Clin Epidemiol. 2019;110:12–22. doi: 10.1016/j.jclinepi.2019.02.004 30763612

[36] Bengio Y, Simard P, Frasconi P. Learning long-term dependencies with gradient descent is difficult. IEEE Trans Neural Netw. 1994;5(2):157–66. doi: 10.1109/72.279181 18267787

[37] Vaswani A, Shazeer N, Parmar N, Uszkoreit J, Jones L, Gomez AN, et al. Attention is all you need. Advances in Neural Information Processing Systems. 2017;30:2017.

[38] Devlin J, Chang MW, Lee K, Toutanova K. BERT: Pre-training of Deep Bidirectional Transformers for Language Understanding. In: Proceedings of the Conference of the North American Chapter of the Association for Computational Linguistics: Human Language Technologies. 2019. p. 4171–86. https://arxiv.org/abs/1810.04805v2

[39] Alsentzer E, Murphy JR, Boag W, Weng WH, Jin D, Naumann T, et al. Publicly available clinical BERT embeddings. arXiv preprint 2019. https://arxiv.org/abs/1904.03323v3

[40] Huang K, Altosaar J, Ranganath R. ClinicalBERT: modeling clinical notes, predicting hospital readmission. In: CHIL ’20: ACM Conference on Health, Inference and Learning; Workshop Track; 2020 Apr 02–04; Toronto, ON. 2020. [cited 2024 July 10]. https://arxiv.org/abs/1904.05342v3

[41] Nazyrova N, Chahed S, Chausalet T, Dwek M. Leveraging large language models for medical text classification: a hospital readmission prediction case. In: 2024 14th International Conference on Pattern Recognition Systems (ICPRS). IEEE; 2024. p. 1–7. 10.1109/icprs62101.2024.10677826

[42] Lee J, Yoon W, Kim S, Kim D, Kim S, So CH, et al. BioBERT: a pre-trained biomedical language representation model for biomedical text mining. Bioinformatics. 2020;36(4):1234–40. doi: 10.1093/bioinformatics/btz682 31501885 PMC7703786

[43] Beltagy I, Lo K, Cohan A. SciBERT: a pretrained language model for scientific text. arXiv preprint 2019. https://arxiv.org/abs/1903.10676v3

[44] Tayefi M, Ngo P, Chomutare T, Dalianis H, Salvi E, Budrionis A, et al. Challenges and opportunities beyond structured data in analysis of electronic health records. WIREs Computational Stats. 2021;13(6). doi: 10.1002/wics.1549

[45] Zhang D, Yin C, Zeng J, Yuan X, Zhang P. Combining structured and unstructured data for predictive models: a deep learning approach. BMC Med Inform Decis Mak. 2020;20(1):280. doi: 10.1186/s12911-020-01297-6 33121479 PMC7596962

[46] Lin Y-W, Zhou Y, Faghri F, Shaw MJ, Campbell RH. Analysis and prediction of unplanned intensive care unit readmission using recurrent neural networks with long short-term memory. PLoS One. 2019;14(7):e0218942. doi: 10.1371/journal.pone.0218942 31283759 PMC6613707

[47] Rajkomar A, Oren E, Chen K, Dai AM, Hajaj N, Hardt M, et al. Scalable and accurate deep learning with electronic health records. NPJ Digit Med. 2018;1:18. doi: 10.1038/s41746-018-0029-1 31304302 PMC6550175

[48] Johnson AEW, Bulgarelli L, Shen L, Gayles A, Shammout A, Horng S, et al. MIMIC-IV, a freely accessible electronic health record dataset. Sci Data. 2023;10(1):1. doi: 10.1038/s41597-022-01899-x 36596836 PMC9810617

[49] Davis S, Zhang J, Lee I, Rezaei M, Greiner R, McAlister FA, et al. Effective hospital readmission prediction models using machine-learned features. BMC Health Serv Res. 2022;22(1):1415. doi: 10.1186/s12913-022-08748-y 36434628 PMC9700920

[50] Raffel C, Shazeer N, Roberts A, Lee K, Narang S, Matena M, et al. Exploring the limits of transfer learning with a unified text-to-text transformer. Journal of Machine Learning Research. 2020;21(140):1–67.34305477

[51] Johnson AEW, Bulgarelli L, Shen L, Gayles A, Shammout A, Horng S, et al. MIMIC-IV, a freely accessible electronic health record dataset. Sci Data. 2023;10(1):1. doi: 10.1038/s41597-022-01899-x 36596836 PMC9810617

[52] Goldberger AL, Amaral LA, Glass L, Hausdorff JM, Ivanov PC, Mark RG, et al. PhysioBank, PhysioToolkit, and PhysioNet: components of a new research resource for complex physiologic signals. Circulation. 2000;101(23):E215-20. doi: 10.1161/01.cir.101.23.e215 10851218

[53] Gu Y, Tinn R, Cheng H, Lucas M, Usuyama N, Liu X, et al. Domain-specific language model pretraining for biomedical natural language processing. ACM Trans Comput Healthcare. 2021;3(1):1–23. doi: 10.1145/3458754

[54] Deka P, Jurek-Loughrey A, P D. Multiple Evidence Combination for Fact-Checking of Health-Related Information. In: The 22nd Workshop on Biomedical Natural Language Processing and BioNLP Shared Tasks, 2023. 237–47. 10.18653/v1/2023.bionlp-1.20

[55] Williams A, Nangia N, Bowman SR. A broad-coverage challenge corpus for sentence understanding through inference. arXiv preprint 2017. https://arxiv.org/abs/1704.05426

[56] Shivade C. MedNLI: A natural language inference dataset for the clinical domain. In: Proceedings of the 2018 Conference on Empirical Methods in Natural Language Processing. Brussels, Belgium: Association for Computational Linguistics; 2019. p. 1586–96.

[57] Pedregosa F, Varoquaux G, Gramfort A, Michel V, Thirion B, Grisel O. Scikit-learn: machine learning in python. Journal of Machine Learning Research. 2011;12:2825–30.

[58] Paszke A, Gross S, Massa F, Lerer A, Bradbury J, Chanan G, et al. PyTorch: an imperative style, high-performance deep learning library. Advances in Neural Information Processing Systems. 2019;32.

[59] Fawcett T. An introduction to ROC analysis. Pattern Recognition Letters. 2006;27(8):861–74. doi: 10.1016/j.patrec.2005.10.010

[60] Matthews BW. Comparison of the predicted and observed secondary structure of T4 phage lysozyme. Biochim Biophys Acta. 1975;405(2):442–51. doi: 10.1016/0005-2795(75)90109-9 1180967

[61] Fensore C, Carrillo-Larco RM, Patel SA, Morris AA, Ho JC. Large language models for integrating social determinant of health data: a case study on heart failure 30-day readmission prediction. arXiv preprint 2024. https://arxiv.org/abs/2407.09688v1

[62] Wolf T, Debut L, Sanh V, Chaumond J, Delangue C, Moi A, et al. Huggingface’s transformers: state-of-the-art natural language processing. arXiv preprint 2020. https://arxiv.org/abs/1910.03771v5

