# Supplementary material for: Predicting 30-day hospital readmissions using ClinicalT5 with structured and unstructured electronic health records
Source: PLoS One. 2025 Sep 2;20(9):e0328848. doi: 10.1371/journal.pone.0328848 (PMC12404500; doi:10.1371/journal.pone.0328848)
Supplement: S1 - Guidelines Accessing Dataset — (DOCX) [file pone.0328848.s001.docx]

**Predicting 30-Day Hospital Readmissions Using ClinicalT5 with Structured and Unstructured Electronic Health Records**

**Dataset & Model**

This study utilizes data from the Medical Information Mart for Intensive Care IV (MIMIC-IV) dataset. Due to ethical constraints, the MIMIC data cannot be shared publicly. Although de-identified, the data contains sensitive information and is subject to access restrictions. However, researchers who have completed the required human research training and signed a data use agreement may request access to the data via PhysioNet.

**Dataset:**

The following three requirements must be met in order to gain access:

- Completion of [CITI Data or Specimens Only Research](https://physionet.org/content/mimiciv/view-required-training/3.1/#1) training
- Become a [Credentialed user on PhysioNet](https://physionet.org/settings/credentialing/)
- Sign the Data Use Agreement specific to the [MIMIC-IV](https://physionet.org/content/mimiciv/2.2/) dataset

Once these requirements are fulfilled, access to the dataset is granted, and data can be accessed either through Google BigQuery or by downloading it locally. For further guidance, please refer to the project website: <https://mimic.mit.edu>.

**Model:**

The large language model (LLM) used to build the hybrid model is Clinical-T5, specifically the Clinical-T5-Scratch variant, which employs the same architecture as T5-Base with 220 million parameters.

Access to this model is granted in the same manner as described above, via PhysioNet. After completing the required training, the Data Use Agreement can be signed at <https://physionet.org/content/clinical-t5/1.0.0/>, where access to the model is also provided.
